# Evolving non-invasive biomarkers in NSCLC immunotherapy: integrating liquid biopsy and multi-omics profiling for precision oncology

**DOI:** 10.3389/fimmu.2026.1900900

**Published:** 2026-07-28

**Authors:** Qiaoyi Shen, Yibo Gao

**Affiliations:** Department of Thoracic Surgery, National Cancer Center/National Clinical Research Center for Cancer/Cancer Hospital, Chinese Academy of Medical Sciences and Peking Union Medical College, Beijing, China

**Keywords:** artificial intelligence, immunotherapy, liquid biopsy, multi-omics, non-small cell lung cancer, precision oncology

## Abstract

The therapeutic landscape for advanced non-small cell lung cancer (NSCLC) has been transformed by immune checkpoint inhibitors (ICIs), yet significant response heterogeneity necessitates robust, dynamic predictive biomarkers. Conventional tissue-based markers, such as PD-L1 expression and tumor mutational burden (TMB), are hindered by their invasive nature and inability to capture the dynamic tumor-host immune interplay. This review synthesizes the paradigm shift toward a dynamic, multi-parametric framework for precision immuno-oncology. We highlight the clinical utility of the liquid biopsy toolbox—including circulating tumor DNA (ctDNA) for molecular residual disease (MRD) monitoring, circulating tumor cells (CTCs), and extracellular vesicles (EVs) in reflecting systemic immune status. Furthermore, we explore biological insights from multi-omics profiling, covering genomic drivers of resistance (e.g., STK11/KEAP1), immunometabolic crosstalk, and systemic inflammatory indicators like the NLR/PLR ratio. The potential of radiomics and pathomics to extract spatial signatures via artificial intelligence (AI) is discussed to address whole-tumor heterogeneity. Finally, we emphasize the integration of these disparate data streams through multimodal AI and Explainable AI (XAI) to construct high-fidelity predictive models. This integrated approach aims to overcome standardization hurdles and enable personalized, adaptive management in NSCLC immunotherapy.

## 1 Introduction: the unmet need for precision in NSCLC immunotherapy

Lung cancer remains the leading cause of cancer-related mortality worldwide, with non-small cell lung cancer (NSCLC) accounting for approximately 85% of all cases (1, 2). The therapeutic landscape for advanced NSCLC has undergone a paradigm shift over the past decade, driven largely by the remarkable success of immune checkpoint inhibitors (ICIs) targeting the programmed death-1 (PD-1) and programmed death-ligand 1 (PD-L1) axis (2, 3). Landmark clinical trials, including KEYNOTE-024, KEYNOTE-042, CheckMate 057, and POPLAR, have established ICIs as a cornerstone of first-line and subsequent therapy for patients without actionable driver mutations, demonstrating significant improvements in overall survival (OS) and durable responses in a subset of patients (4–8). Despite these advances, only a fraction of patients derive clinical benefit, with primary resistance observed in 50-75% of cases and acquired resistance emerging even among initial responders (9, 10).

The challenge of patient selection is compounded by the limitations of existing biomarkers. PD-L1 expression, assessed by immunohistochemistry, has been the most widely adopted predictive biomarker, yet its utility is constrained by temporal and spatial heterogeneity, variable assay platforms, and inconsistent cutoffs across tumor types and treatment contexts (11–14). Specifically, spatial heterogeneity manifests as discordant PD-L1 expression not only within different regions of the same primary tumor (intra-tumoral) but also between the primary tumor and paired metastatic sites (inter-tumoral) (15). Clinical studies have reported PD-L1 discordance rates ranging from 20% to nearly 50% between matched primary and metastatic lesions, potentially leading to misclassification if only a single biopsy is evaluated (16). Furthermore, temporal heterogeneity significantly complicates longitudinal management, as interventions like chemotherapy or targeted therapies can dynamically upregulate or downregulate PD-L1 status. Thus, a historical baseline biopsy may fail to accurately reflect the tumor’s immune profile at the time of disease progression or subsequent lines of therapy (17). A comprehensive analysis of all US Food and Drug Administration approvals of ICIs revealed that PD-L1 expression was predictive in only 28.9% of cases, underscoring its inadequacy as a standalone biomarker (11). Tumor mutational burden (TMB) has emerged as an alternative, with prospective data from the KEYNOTE-158 study demonstrating that high tissue TMB (≥10 mutations per megabase) identifies patients with enhanced response to pembrolizumab across multiple solid tumors (18). However, the association between TMB and clinical benefit is not uniform across all cancer types, and the correlation between TMB and PD-L1 expression remains weak, suggesting that these biomarkers capture distinct aspects of tumor immunogenicity (19, 20). More importantly, the broad clinical utility of tissue TMB is hindered by a lack of standardization across different targeted sequencing platforms and the absence of universally accepted optimal cutoffs (21). Biologically, TMB primarily quantifies the absolute number of mutations but fails to account for the “quality” or immunogenicity of these mutations. For instance, it does not distinguish between clonal mutations—which are present in all tumor cells and generate widely presented neoantigens that elicit robust anti-tumor immunity—and subclonal mutations, which are confined to specific tumor branches and are less effective at driving a systemic immune response (22). Microsatellite instability (MSI) represents another FDA-approved biomarker, though its prevalence in NSCLC is low, limiting its clinical applicability (23, 24).

The emergence of liquid biopsy has opened new avenues for overcoming the inherent limitations of tissue-based biomarkers, including the challenges of tumor heterogeneity, insufficient tissue availability, and the inability to perform real-time monitoring (25, 26). Circulating tumor DNA (ctDNA), circulating tumor cells (CTCs), and EVs each offer unique advantages for capturing the dynamic genomic and epigenomic landscape of tumors throughout the course of immunotherapy (25, 27, 28). Concurrently, advances in multi-omics profiling—encompassing genomics, epigenomics, transcriptomics, and fragmentomics—have enabled comprehensive characterization of the tumor-immune interface at unprecedented resolution (29, 30). The integration of these approaches through machine learning frameworks holds promise for developing robust, non-invasive biomarkers that can guide treatment decisions, predict outcomes, and enable early detection of resistance (29). Moreover, recent methodological breakthroughs and expanded molecular profiling strategies have further enriched the translational potential of these biomarkers, offering unprecedented precision in capturing dynamic tumor evolution (31–33).

## 2 The expanding liquid biopsy toolbox for immunotherapy monitoring

### 2.1 Circulating tumor DNA: dynamics, clearance, and MRD

Circulating tumor DNA, comprising fragmented DNA released into the bloodstream by apoptotic or necrotic tumor cells, has emerged as one of the most clinically advanced liquid biopsy analytes in oncology (34, 35). In the context of NSCLC immunotherapy, ctDNA analysis offers a minimally invasive window into tumor genetics, burden dynamics, and clonal evolution that is increasingly recognized as a valuable tool for predicting and monitoring treatment response (36, 37). The clinical utility of ctDNA is rooted in its ability to reflect the real-time molecular state of the tumor, providing information that complements and often surpasses that obtained from static tissue biopsies (38, 39).

The predictive value of baseline ctDNA levels for immunotherapy outcomes has been a subject of extensive investigation. A systematic review and meta-analysis encompassing over 1000 patients with advanced NSCLC treated with ICIs found that pre-treatment ctDNA detection status alone was not significantly associated with OS or progression-free survival (PFS) (40). This finding highlights the limitation of a single time-point measurement, as ctDNA levels are influenced by multiple factors including tumor burden, histology, and the rate of cell turnover (41). However, the same meta-analysis revealed that early longitudinal assessment of ctDNA dynamics provides robust prognostic information. Specifically, based on comprehensive meta-analyses and recent expert consensus, a reduction in ctDNA concentration early during treatment—typically within the first 6 to 12 weeks—was strongly associated with significant improvements in OS (hazard ratio [HR], 0.19), PFS (HR, 0.30), and objective response rate (ORR) (28, 40). Importantly, as a reference for real-world clinical practice, if combined with concurrent radiographic imaging assessments, this evaluation time window for early dynamic changes can be appropriately shortened. This early molecular response, often preceding radiographic changes by weeks to months, represents a powerful tool for distinguishing true responders from non-responders (35, 42).

The concept of ctDNA clearance, defined as the elimination of detectable ctDNA from the circulation, has garnered particular attention as a surrogate for deep and durable treatment response. In patients with advanced NSCLC receiving PD-(L)1 blockade, ctDNA clearance during treatment correlates strongly with improved clinical outcomes and sustained disease control (43, 44). Conversely, the persistence or emergence of ctDNA during therapy is highly indicative of disease progression and treatment resistance (25, 35). The ability to detect MRD through ctDNA analysis is also gaining traction in the perioperative setting for early-stage NSCLC (45, 46). In patients undergoing neoadjuvant chemo-immunotherapy, ctDNA clearance before surgery is associated with higher rates of major pathological response (MPR) and pathological complete response (pCR) (46, 47). Postoperatively, landmark ctDNA positivity identifies patients at extremely high risk of disease recurrence, often preceding radiographic detection by several months (45, 48). This capability to identify MRD offers the potential to guide adjuvant therapy decisions and tailor surveillance strategies for individual patients (47, 49).

Beyond simple quantification, the genomic characterization of ctDNA provides additional layers of predictive information. The assessment of blood-based TMB (bTMB) has been explored as a non-invasive alternative to tissue TMB (tTMB) (37, 50). In the phase 2 B-F1RST trial, which prospectively evaluated bTMB as a predictive biomarker for first-line atezolizumab in advanced NSCLC, a higher bTMB (≥16 mutations per megabase) was associated with improved ORR and, at longer follow-up, prolonged OS (50). However, the concordance between bTMB and tTMB is not perfect, and bTMB may reflect a composite index that encompasses both tumor heterogeneity and circulating tumor DNA fraction, rather than being a direct substitute for tissue-based assessment (51, 52). Emerging evidence also highlights the utility of ctDNA fragmentomic analysis, including the assessment of fragment length profiles and copy number variations (CNVs), as complementary indicators of tumor biology and treatment response. Low-coverage whole-genome sequencing (lcWGS) of ctDNA has demonstrated that aberrant fragmentation patterns at baseline are significantly associated with subsequent PFS and can improve predictions based on PD-L1 expression alone (53). The integrative analysis of ctDNA fragment characteristics, tumor PD-L1 expression, and residual ctDNA under treatment constitutes a strong independent predictor of both PFS and OS (53).

### 2.2 CTCs and EVs: uncovering phenotypic and functional heterogeneity

While ctDNA provides a wealth of genomic information, it is limited in its ability to reveal the phenotypic and functional heterogeneity of tumor cells that is critical for understanding immune evasion and therapeutic resistance (54, 55). CTCs, intact cancer cells shed into the bloodstream from primary or metastatic sites, offer a unique opportunity to study the cellular characteristics of the disseminating tumor (54, 56). The analysis of CTCs in the context of immunotherapy is particularly intriguing due to the potential to assess the expression of immune checkpoint proteins on their surface, such as PD-L1 (57, 58). Studies have shown that the presence and number of PD-L1-positive CTCs in the blood of patients with NSCLC are associated with poor OS and treatment failure, suggesting that these cells may represent an aggressive, immune-evasive subpopulation (58, 59). Furthermore, the phenotypic heterogeneity of CTCs, including the co-expression of epithelial and mesenchymal markers indicative of partial epithelial-mesenchymal transition (EMT), has been linked to stemness and increased metastatic potential (54). Tracking these dynamic phenotypic shifts in CTCs during ICI therapy could provide early insights into the emergence of resistance mechanisms. However, the successful clinical application of CTC analysis heavily relies on the detection methodologies employed. The widely used, FDA-cleared CellSearch system utilizes EpCAM-based immunomagnetic bead sorting (60, 61); while highly standardized, its sensitivity is often compromised in NSCLC because it fails to capture CTCs that have undergone EMT and consequently lost epithelial markers (62). To overcome this biological bias, advanced label-free microfluidic chips (which isolate cells based on physical properties like size and deformability) and next-generation multiplexed immunomagnetic sorting (incorporating both epithelial and mesenchymal markers) have been developed (63). These newer technologies exhibit significantly higher sensitivity for capturing heterogeneous CTC subpopulations, including mesenchymal-like CTCs (64). Consequently, the choice of detection platform directly impacts the detection rate of PD-L1-positive CTCs and the resulting predictive readouts, highlighting the critical need to align the appropriate technology with the specific clinical application (65).

EVs, particularly exosomes, have emerged as another critical component of the liquid biopsy toolbox, offering a rich source of molecular information that reflects the state of their parental cells (55, 66). These small, membrane-bound particles are actively secreted by all cell types, including tumor cells, and carry a diverse cargo of proteins, lipids, and nucleic acids, including messenger RNA, microRNA (miRNA), and long non-coding RNA (lncRNA) (66–68). The role of EVs in mediating intercellular communication within the tumor microenvironment (TME) is increasingly recognized, and their cargo can provide valuable insights into tumor-immune interactions and therapeutic resistance (69, 70).

One of the most extensively studied EV-associated biomarkers in NSCLC immunotherapy is exosomal PD-L1. Tumor-derived exosomes carry functional PD-L1 on their surface, which can directly bind to PD-1 on T cells, inhibiting their cytotoxic activity and contributing to systemic immune suppression (69, 71). The dynamics of exosomal PD-L1 in the blood during ICI treatment have shown significant promise as a predictive biomarker. A study analyzing EV PD-L1 dynamics in two independent cohorts of patients with advanced NSCLC found that an increase in EV PD-L1 levels at 9 weeks compared to baseline was observed in non-responders and was an independent biomarker for shorter PFS and OS (44). While tissue PD-L1 expression did not predict durable response in this specific analysis (44), it is important to interpret these findings cautiously, as such conclusions may be limited by the study’s sample size and specific cohort characteristics. Tissue PD-L1 unequivocally remains the standard-of-care routine clinical test and cannot be entirely replaced at present. Therefore, rather than claiming superiority, dynamic blood-based EV PD-L1 monitoring should be viewed as a highly valuable complementary tool—one that compensates for tissue spatial limitations by capturing real-time systemic immune evasion during treatment.

Beyond PD-L1, the nucleic acid cargo of EVs, particularly miRNAs and lncRNAs, is being actively investigated for its predictive and prognostic value (67, 72). Specific EV-associated miRNAs have been linked to immunotherapy response. For instance, a study profiling plasma EV miRNAs in patients with metastatic NSCLC and high PD-L1 expression found that elevated levels of miR-106b, miR-451a, miR-181a, and miR-10a in pre-treatment serum EVs were significantly associated with a lack of response to first-line pembrolizumab (72). Low levels of these miRNAs also predicted improved PFS, with miR-106b additionally correlating with longer OS (72). Similarly, the lncRNA H19, identified in plasma cell-free RNA, has been associated with disease progression (67). The comprehensive analysis of the EV circular RNA (circRNA) landscape across various human biofluids has also revealed cancer-associated circRNAs linked to clinical outcomes, including the potential to serve as biomarkers for evaluating immunotherapy efficacy in NSCLC (68). The integration of these diverse EV-encapsulated biomarkers, often analyzed using multi-omics approaches, is paving the way for more holistic and informative liquid biopsy panels (73, 74).

### 2.3 Soluble immune and inflammatory mediators: systemic signatures of response and toxicity

The systemic immune response to immunotherapy is not confined to the tumor itself but is reflected in the composition of circulating immune cells and soluble factors in the peripheral blood (58, 75). These readily accessible biomarkers offer a window into the host’s immune status and its dynamic modulation by ICI therapy (76, 77). Complete blood counts and derived ratios, such as the neutrophil-to-lymphocyte ratio (NLR), the platelet-to-lymphocyte ratio (PLR), and the derived NLR, have been extensively studied as prognostic and predictive markers in NSCLC patients treated with ICIs (77, 78). A high baseline NLR, reflecting a state of systemic inflammation and relative lymphopenia, is generally associated with poorer outcomes, including shorter PFS and OS (77, 79). These inexpensive and widely available parameters can be easily integrated into clinical practice to provide an initial risk stratification.

More detailed immunophenotyping of peripheral blood mononuclear cells (PBMCs) by flow cytometry provides a deeper understanding of the cellular dynamics underlying treatment response (57, 80). The frequencies and activation states of various immune cell subsets, including CD8+ and CD4+ T cells, regulatory T cells (Tregs), natural killer (NK) cells, and myeloid-derived suppressor cells (MDSCs), have all been implicated in modulating ICI efficacy (58, 75). For example, a high frequency of PD-1-positive CD8+ T cells in the blood has been associated with favorable outcomes, while a high count of PD-1-positive CD4+ T cells has been linked to poor survival (58). The analysis of T-cell receptor (TCR) repertoire diversity and clonality in the blood is another promising approach, as an expanding and diverse T-cell repertoire during treatment is thought to reflect a robust antitumor immune response (57, 59). Studies have shown that increased clonality and the emergence of new T-cell clones in the periphery can correlate with clinical benefit from ICI therapy (57).

Soluble factors in the plasma, including cytokines, chemokines, and soluble immune checkpoint molecules, constitute another layer of systemic biomarkers (81, 82). Pre-treatment levels of soluble PD-L1 (sPD-L1) have been investigated, with some studies suggesting that low baseline levels are predictive of better treatment responses (58, 77). However, the data remain conflicting, and the biological significance of sPD-L1, which can be shed from both tumor and immune cells, is not fully understood (82). A more consistent finding is the association of certain soluble inflammatory mediators with both treatment response and the development of immune-related adverse events (irAEs) (83, 84). For instance, elevated levels of specific chemokines like CXCL9 and CXCL10, which are involved in T-cell trafficking, have been linked to favorable ICI outcomes (73). Conversely, increases in acute-phase proteins and certain cytokines may herald the onset of systemic inflammation and irAEs (79, 83). The identification of blood-based signatures that can simultaneously predict efficacy and toxicity is a major goal of current research, as it would enable a more balanced and patient-centered approach to ICI therapy (83, 85).

## 3 Multi-omics profiling for deeper biological insights

### 3.1 Genomic determinants and TMB: from tissue to blood

The genomic landscape of a tumor is a primary determinant of its immunogenicity and its susceptibility to ICI therapy (86, 87). While PD-L1 expression and TMB are the most established genomic biomarkers, a deeper understanding of specific gene mutations and their impact on the TME is refining patient stratification (88, 89). The assessment of TMB has evolved from tissue-based whole-exome sequencing to targeted gene panels, and more recently, to bTMB (90, 91). Despite the promise of bTMB as a non-invasive tool, its clinical utility is still being defined. The B-F1RST trial provided prospective evidence for its predictive value, but challenges remain regarding assay standardization, threshold definition, and the impact of clonal hematopoiesis of indeterminate potential (CHIP) on bTMB calculation (50, 52). A critical distinction is that bTMB is not identical to tTMB; it may reflect not only the mutational load of the primary tumor but also contributions from heterogeneous metastatic sites and the overall ctDNA burden (51).

**Figure 1:**
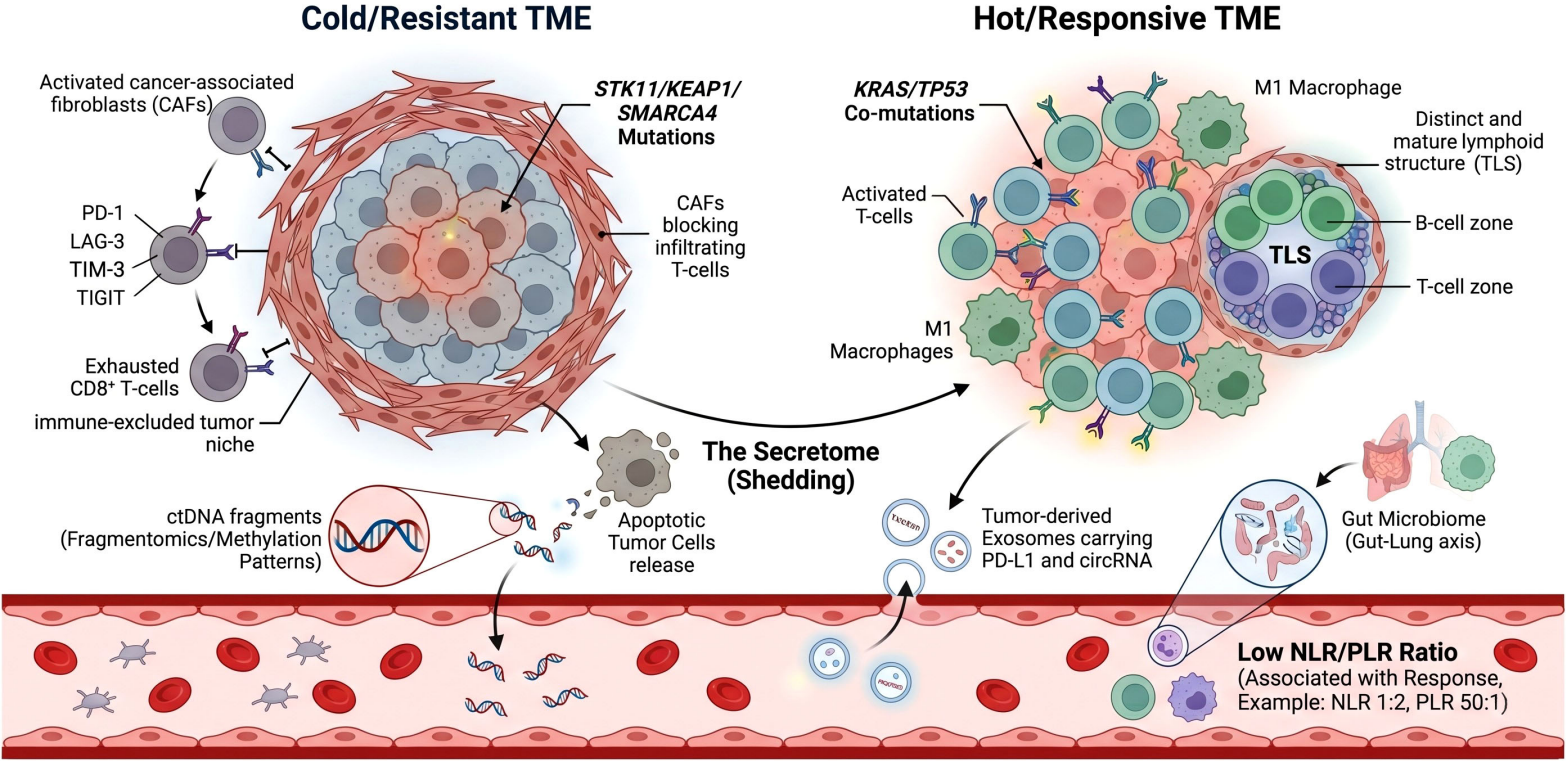
The interplay between tumor genomics and systemic biomarkers. Cold/Resistant TME: Often driven by STK11, KEAP1, or SMARCA4 mutations, this “immune-excluded” phenotype is characterized by activated cancer-associated fibroblasts (CAFs) that form a physical barrier, blocking T-cell infiltration. Peripheral or excluded CD8+ T cells exhibit exhaustion programs, co-expressing inhibitory receptors such as PD-1, LAG-3, TIM-3, and TIGIT. Hot/Responsive TME: Frequently associated with KRAS/TP53 co-mutations, this “inflamed” niche features infiltrating M1 macrophages and mature TLS. These TLS display distinct and organized B-cell and T-cell zones, which are associated with favorable outcomes. Systemic Biomarkers: Intratumoral states are reflected systemically via The Secretome (Shedding), including ctDNA fragments with distinct fragmentomics and methylation patterns, as well as tumor-derived exosomes carrying functional PD-L1 and circRNA. Additionally, a low NLR/PLR ratio and a healthy Gut Microbiome (via the gut-lung axis) serve as systemic indicators of a favorable immune response.

Beyond quantitative TMB, the qualitative nature of specific genomic alterations profoundly influences the immune microenvironment and ICI response (86, 89). Mutations in the tumor suppressor gene STK11 (LKB1) and the oxidative stress response gene KEAP1 are among the most potent drivers of primary resistance to immunotherapy in NSCLC (86, 92). These mutations, which often co-occur with KRAS mutations, are associated with an immune-excluded or cold TME characterized by low T-cell infiltration, reduced antigen presentation, and a paucity of tertiary lymphoid structures (TLS) (86, 89). The presence of STK11 or KEAP1 mutations confers a poor prognosis independent of PD-L1 expression or TMB, making them powerful exclusionary biomarkers for ICI benefit (86, 88, 92). Similarly, mutations in SMARCA4, a component of the SWI/SNF chromatin remodeling complex, lead to impaired antigen presentation and a poorly immunogenic tumor phenotype, also contributing to ICI resistance (86). In contrast, co-mutations in KRAS and TP53 are often associated with a more inflamed, immune-hot TME, characterized by higher TMB, increased CD8+ T-cell infiltration, and better responses to ICIs (73, 89) (**Figure 1**). The presence of actionable driver mutations, such as EGFR, ALK, and ROS1, is generally associated with lower TMB and an immunosuppressive TME, traditionally leading to intrinsic resistance to ICI monotherapy (93, 94). However, absolute statements regarding immunotherapy resistance in these populations should be avoided, as emerging clinical evidence highlights notable exceptions. For instance, preliminary data suggest that patients harboring specific alterations, such as EGFR exon 20 insertions, or those with ALK fusions may derive significant clinical benefit from ICIs when combined with anti-angiogenic therapy and chemotherapy (95). Furthermore, patients with EGFR-mutant NSCLC and high PD-L1 expression may represent a biologically distinct subtype with attenuated oncogene addiction and a dysfunctional TME, where ICI benefit is still uncertain and requires careful integration with targeted therapy (94).

### 3.2 Proteomics and metabolomics: unraveling the immunometabolic crosstalk

Genomics and transcriptomics provide information about the potential of the tumor and immune system, but they do not directly capture the functional state of proteins and metabolites, which are the ultimate effectors of biological processes (73, 96). Proteomic and metabolomic profiling of liquid biopsies, particularly serum or plasma, offers a direct readout of the functional and metabolic status of the patient, revealing pathways that are actively dysregulated in the context of immunotherapy (73, 83). Mass spectrometry-based serum proteomics has led to the development of multi-analyte tests that can stratify patients into good and poor outcome groups independent of PD-L1 expression (83). For instance, the PROphet test, which measures a panel of serum proteins, has demonstrated the ability to predict ICI response in both NSCLC and melanoma patients (83). These proteomic signatures often encompass components of the complement cascade, acute-phase proteins, and chemokines, reflecting systemic inflammation and immune activation (73, 83, 97).

The integration of proteomics with lipidomics and metabolomics is revealing the critical role of immunometabolic crosstalk in shaping ICI response (97, 98). The tumor microenvironment is a metabolically hostile niche where competition for nutrients and the accumulation of immunosuppressive metabolites can cripple effector T-cell function (98). An integrated lipidomic and proteomic analysis of pre-treatment serum from NSCLC patients treated with nivolumab identified lysophosphatidylcholine (LPC), particularly LPC(20:0), as a promising predictive biomarker, with elevated levels in responders (97). This LPC signature is thought to reflect a systemic immunometabolic state involving platelet and neutrophil activity (97). Conversely, metabolic profiling has linked ICI resistance to increased levels of immunosuppressive metabolites such as kynurenine, a product of the indoleamine 2,3-dioxygenase (IDO) pathway, and elevated lactate, which can impair T-cell function and promote regulatory immune subsets (73, 98). The identification of specific metabolic vulnerabilities, such as glutamine dependence or adenosine signaling, opens up new avenues for combining metabolic inhibitors with ICIs to overcome resistance (98). The ongoing refinement of metabolomic and lipidomic platforms, coupled with their integration into multi-omics frameworks, is essential for translating these complex metabolic signatures into clinically actionable biomarkers (73, 98).

### 3.3 Transcriptomics and the tumor immune microenvironment: cell-type and spatial context

The tumor immune microenvironment, a complex ecosystem of malignant cells, immune cells, stromal cells, and extracellular matrix, is the central battleground where the fate of immunotherapy is decided (99, 100). Transcriptomic profiling, particularly at the single-cell and spatial level, has revolutionized our understanding of the TIME, revealing its remarkable heterogeneity and dynamic evolution (99, 101, 102). Single-cell RNA sequencing (scRNA-seq) allows for the unbiased identification of all cell types within a tumor, including rare subsets, and can delineate their functional states, such as exhaustion, activation, or suppression (101, 103). This technology has been instrumental in mapping the transcriptional landscape of tumor-infiltrating lymphocytes (TILs) in NSCLC, identifying specific CD8+ T-cell exhaustion programs associated with ICI resistance (104). A 25-gene signature indicative of CD8+ T-cell exhaustion, characterized by the co-expression of multiple immune checkpoints like PD-1, LAG-3, TIGIT, and TIM-3, has been shown to predict ICI responsiveness with high accuracy (104).

Spatial transcriptomics adds a critical dimension by preserving the geographical context of gene expression within the tissue, allowing for the visualization of cellular interactions and the organization of immune niches (105, 106). This approach has been particularly powerful in studying TLS, which are organized aggregates of B and T cells that form at the tumor site and are associated with favorable ICI outcomes (106, 107). However, not all TLS-positive tumors respond to ICI. Spatial transcriptomics has revealed that the presence of specific subsets of cancer-associated fibroblasts (CAFs) within the TME can mediate primary resistance even in the presence of mature TLS (106). These immunosuppressive CAFs are associated with immune exclusion and CD8+ T-cell exhaustion, highlighting the complex interplay between different stromal components and immune cells (106). A comprehensive spatial multi-omics approach integrating spatial proteomics and transcriptomics has identified robust cell-type signatures for both resistance (including proliferating tumor cells, granulocytes, and vessels) and response (including M1/M2 macrophages and CD4+ T cells) (105). These spatial signatures were predictive of clinical outcomes across multiple independent cohorts, underscoring their translational potential (105).

The integration of transcriptomic data with other omics layers is crucial for building a holistic picture of the TIME. The B-cell-autoantibody axis, often overlooked in the T-cell-centric view of immunotherapy, has emerged as a critical component of the antitumor immune response (107). Tumor-infiltrating B lymphocytes (TIL-Bs) and the autoantibodies they produce can contribute to tumor control through antibody-dependent cell-mediated cytotoxicity (ADCC) and complement activation, but they can also promote immune suppression (107). The maturation status of TLS dictates the functional plasticity of TIL-Bs, switching between anti-tumor effector phenotypes and pro-tumor regulatory roles (107). Understanding the dualistic role of this axis is crucial for developing next-generation immunotherapies, including B-cell-based vaccines and strategies to modulate TLS neogenesis (107). LncRNAs have also emerged as critical regulators of the TIME, modulating immune evasion through PD-1/PD-L1 signaling, influencing T-cell survival, and affecting macrophage phagocytic checkpoints (108). Their expression in exosomes offers a minimally invasive tool for liquid biopsy and longitudinal monitoring (108).

## 4 Integrating modalities: the rise of multimodal and artificial intelligence models

### 4.1 Radiomics and pathomics: imaging and spatial signatures of immune response

While molecular biomarkers provide profound insights, they are inherently limited by sampling bias and the inability to capture the full spatial heterogeneity of the entire tumor burden (109, 110). Medical imaging, particularly computed tomography (CT) and positron emission tomography (PET/CT), offers a non-invasive, whole-tumor perspective that can be quantitatively mined using radiomics (109, 111). Radiomics involves the extraction of a large number of quantitative features from medical images, including texture, shape, and intensity, which can reflect underlying tumor biology, such as cellular density, hypoxia, and vascularity (110, 112). In the context of NSCLC immunotherapy, radiomic features have shown promise in predicting PD-L1 expression, TMB, and ultimately, treatment response and survival (110, 111, 113). A meta-analysis of CT texture analysis studies found that patients stratified as low-risk based on quantitative CT texture signatures had substantially better OS and PFS compared to high-risk patients (112).

The predictive power of radiomics is often enhanced by incorporating dynamic changes in imaging features during treatment. Delta-radiomics, which quantifies the change in radiomic features between baseline and early follow-up scans, has demonstrated superior performance in distinguishing responders from non-responders compared to single time-point analysis (114). A combined delta-radiomics nomogram incorporating changes in radiomic signatures with clinical factors achieved an area under the curve (AUC) of 0.83 for predicting response, outperforming PD-L1 expression alone (114). PET/CT-based radiomics, which captures both anatomical and metabolic information, provides an even richer dataset. A machine learning model based on PET/CT radiomics and clinical characteristics successfully predicted tumor immune profiles, with the combined model outperforming models based on either modality alone (115). The predicted CD8-high group from this model had significantly higher immune scores and more activated immune pathways, demonstrating the potential of radiomics as a surrogate for invasive immune phenotyping (115).

Pathomics, the application of AI to digitized histopathology slides, is another powerful modality for extracting spatial signatures of the immune response (116, 117). By analyzing the spatial arrangement and morphology of cells in hematoxylin and eosin (H&E) or immunohistochemistry (IHC) stained slides, pathomics can quantify tumor-infiltrating lymphocytes, characterize the tumor-stroma interface, and identify patterns associated with TLS maturation (117, 118). Compared to traditional manual interpretation by pathologists, AI-driven pathomics offers distinct advantages: it enables the high-throughput, objective quantification of lymphocyte density across the entire whole-slide image (119), and possesses the capability to identify subtle morphological features and nascent TLS structures that are often invisible to the naked eye. However, to objectively evaluate its current technical status, several inherent limitations must be acknowledged. The accuracy of pathomics is heavily dependent on the quality and resolution of the scanning equipment, and the models remain susceptible to algorithmic biases stemming from variations in tissue preparation, staining protocols, and the limited diversity of training datasets (120). A multimodal model integrating features from CT scans, digitized PD-L1 IHC slides, and genomic data achieved an AUC of 0.80 for predicting immunotherapy response, significantly outperforming unimodal measures such as TMB (AUC 0.61) and PD-L1 IHC score (AUC 0.73) (117). This study provides a quantitative rationale for using expert-guided machine learning to integrate diverse data types and improve prediction accuracy (117).

### 4.2 Machine learning integration of multi-modal data for enhanced prediction

**Figure 2:**
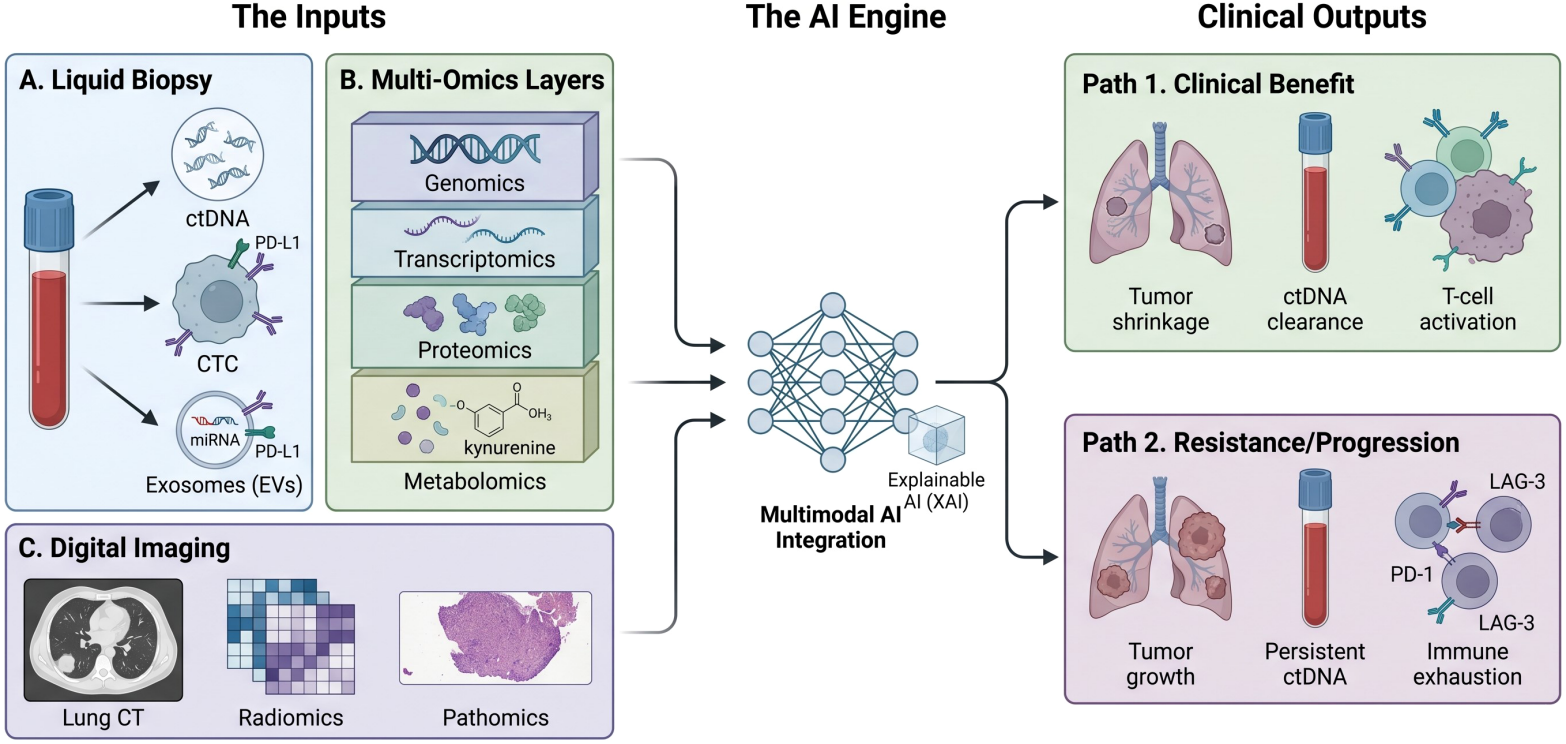
Dynamic, multi-parametric framework for precision immuno-oncology. The Inputs: The framework integrates non-invasive data from three distinct dimensions: **(A)** Liquid Biopsy, capturing circulating tumor DNA (ctDNA), CTCs, and extracellular vesicles (EVs) carrying cargo such as PD-L1 and miRNA; **(B)** Multi-Omics Layers, encompassing genomics, transcriptomics, proteomics, and metabolomics (e.g., kynurenine levels); and **(C)** Digital Imaging, including lung CT scans and derived radiomics and pathomics features. The AI Engine: Multimodal AI integration is utilized for non-linear pattern recognition across disparate data types. Clinical Outputs: Patients are stratified into two primary trajectories: Path 1 (Clinical Benefit), characterized by tumor shrinkage, ctDNA clearance, and robust T-cell activation; and Path 2 (Resistance/Progression), marked by tumor growth, persistent ctDNA, and immune exhaustion, often identified by the co-expression of multiple checkpoints.

The sheer volume and complexity of data generated by liquid biopsy, multi-omics profiling, and imaging necessitate advanced computational approaches for meaningful integration (116, 121). Artificial intelligence, and particularly machine learning, has become indispensable for discovering non-linear relationships and patterns across disparate data modalities that would be impossible to discern through traditional statistical methods (116, 122). Machine learning algorithms can be trained to identify the most informative features from each modality and combine them into a single predictive model that outperforms any individual biomarker (117, 118). This integrative approach is crucial for capturing the multifaceted nature of ICI response, which depends on tumor genomics, host immunity, TME composition, and systemic factors (96, 123) (**Figure 2**). In tandem with these approaches, the continuous optimization of computational algorithms and advanced predictive modeling techniques has shown remarkable utility in decoding complex biomedical datasets, accelerating the paradigm shift toward truly individualized therapeutic strategies (124–126).

Multiple machine learning approaches have been employed to develop robust classifiers for ICI response. For instance, a gene mutation-based predictive signature (GPS) was developed using non-synonymous mutations and multiple machine learning approaches to categorize patients based on their predicted response (127). This GPS classifier outperformed conventional predictors like PD-L1 and TMB when validated in independent cohorts and was further validated using an ex-vivo tumor organoid-PBMC co-culture model (127). Another study used unsupervised machine learning-driven CT radiomic subtypes to stratify NSCLC patients, revealing that one subtype (Cluster 2) had significantly longer survival and was characterized by a more inflamed TME with higher proportions of T cells, B cells, and NK cells (128). This approach demonstrates how unsupervised learning can discover novel, biologically meaningful subgroups directly from imaging data (128).

The integration of multi-omics data is often facilitated by specialized algorithms like Multi-Omics Factor Analysis (MOFA2), which can identify latent molecular subtypes that capture divergent survival outcomes and reflect biologically coherent processes (74). A study using MOFA2 to integrate plasma-derived cell-free DNA (cfDNA) methylation and EV-associated miRNA data from NSCLC patients undergoing ICI therapy identified MOFA-Derived Clusters (MDCs) that were associated with distinct survival outcomes and immune phenotypes (74). These MDC classifiers demonstrated consistent survival stratification in external cohorts, defining a multi-omic, liquid biopsy-based framework for molecular subtyping (74). The development of clinically explainable AI models is also a priority, as clinicians need to understand the rationale behind a prediction to trust and act upon it (118). One study developed a method that learns a graph structure to understand associations between biomarkers and treatment response, which is then used to generate language-based explanations via a Large Language Model (LLM), making the prediction more comprehensible to clinicians (118).

### 4.3 Emerging frontiers: epigenomics, microbiome, and B-cell signatures

Beyond the core modalities of genomics, transcriptomics, proteomics, and imaging, several emerging areas are poised to contribute to a more comprehensive multi-parametric framework for precision immuno-oncology. Epigenomic alterations, including DNA methylation and histone modifications, are dynamic regulators of gene expression that can drive therapeutic resistance (129). The epigenome of tumor cells can be reprogrammed to create an immunosuppressive TME, and conversely, epigenetic drugs can potentially re-sensitize resistant tumors to ICIs (129, 130). The analysis of cfDNA methylation patterns in liquid biopsy offers a non-invasive window into the tumor epigenome (74, 131). A methylation-based ctDNA assay has shown promise for monitoring immunotherapy response, with changes in the tumor methylation score (TMS) strongly correlating with real-world PFS (131). Integrating epigenomic data with other omics layers could provide a more complete picture of the regulatory mechanisms governing ICI response (129).

The gut microbiome has emerged as a key modulator of systemic immunity, capable of influencing the efficacy of ICIs in lung cancer via the gut-lung axis (102, 132). Specific bacterial taxa have been associated with improved ICI outcomes, and composite scoring systems based on microbiome composition are being developed (102, 132). The mechanisms involve microbial metabolites and structural components that can stimulate or suppress antitumor immune responses (132). While methodological heterogeneity in microbiome studies currently limits immediate clinical translation, the field is rapidly advancing from observational research to active clinical intervention. A prime example is the emerging preliminary efficacy data of fecal microbiota transplantation (FMT), which has successfully demonstrated the ability to overcome ICI resistance and re-sensitize refractory patients to immunotherapy (133, 134). These clinical milestones, coupled with innovations in engineered probiotics, establish the targeted modulation of the microbiome as a highly promising therapeutic frontier (132, 135). The integration of microbiome profiles with host and tumor biomarkers represents a truly holistic approach to patient stratification.

Finally, the role of B cells and autoantibodies in the antitumor immune response is being re-evaluated (107). As discussed earlier, TIL-Bs and TLS are critical determinants of ICI response. The analysis of the B-cell receptor (BCR) repertoire and the presence of tumor-specific autoantibodies in the blood offers another avenue for liquid biopsy-based monitoring (107). These B-cell signatures could provide complementary information to T-cell-based biomarkers, particularly in tumors where a robust humoral immune response is a key feature of an inflamed TME (107). The integration of these emerging frontiers into the existing multi-omics and AI-driven framework will be essential for building the next generation of predictive models that can truly capture the full complexity of the host-tumor-immune ecosystem.

## 5 Conclusion and outlook: towards a dynamic, multi-parametric framework for precision immuno-oncology

The quest for precision in NSCLC immunotherapy has driven a paradigm shift from static, tissue-based biomarkers to a dynamic, multi-parametric framework that integrates liquid biopsy, multi-omics profiling, and advanced computational analysis. The evidence reviewed herein demonstrates that no single biomarker is sufficient to capture the complex and heterogeneous nature of ICI response and resistance (123, 136). Instead, the future lies in the intelligent integration of complementary data streams. The liquid biopsy toolbox, encompassing ctDNA, CTCs, EVs, and soluble mediators, provides a minimally invasive and repeatable means to monitor tumor burden, genomic evolution, phenotypic heterogeneity, and systemic immune status in real-time (25, 34). Multi-omics profiling, from genomics and transcriptomics to proteomics and metabolomics, offers deep biological insights into the mechanisms driving response and resistance, revealing the intricate interplay between tumor cell-intrinsic alterations, the TME, and the host immune system (73, 137).

The integration of these diverse modalities is being catalyzed by the rise of artificial intelligence and machine learning. These computational tools are not merely for data reduction; they are essential for discovering non-linear, high-dimensional patterns that can be synthesized into robust predictive models (116, 118). Radiomics and pathomics add the crucial dimension of spatial heterogeneity, capturing whole-tumor and micro-anatomical features that are invisible to molecular assays (109, 117). The convergence of these approaches is leading to the development of composite biomarker panels that consistently outperform any single marker, such as PD-L1 or TMB, in predicting clinical outcomes (105, 117, 127).

Despite these significant advances, several critical challenges must be addressed before these multi-parametric frameworks can be widely adopted in clinical practice. A primary hurdle is the lack of standardization across assays, platforms, and bioinformatics pipelines (102, 138). ctDNA analysis, for example, requires harmonization of pre-analytical variables, sequencing methods, and analytical algorithms to ensure reproducibility across different laboratories and clinical trials (35, 39). Similarly, radiomic features are sensitive to variations in image acquisition and reconstruction parameters, necessitating robust standardization protocols (112, 139). The validation of these complex models in large, prospective, and diverse patient cohorts is paramount to establish their clinical utility and generalizability (116, 139). Many promising biomarkers have been identified in retrospective studies but have failed to demonstrate sufficient predictive accuracy in prospective settings (92, 116).

Another significant challenge is the interpretability of AI-driven models. While a machine learning algorithm may achieve high predictive accuracy, its “black box” nature can be a barrier to clinical acceptance (118, 121). Efforts to develop explainable AI (XAI) that can provide clinicians with understandable rationales for its predictions are crucial for building trust and facilitating integration into decision-making (118). Furthermore, the economic and logistical hurdles of implementing comprehensive multi-omic and AI-based testing in routine clinical care must be considered. The cost of sequencing, proteomic analysis, and advanced imaging, coupled with the need for specialized computational infrastructure and expertise, may limit access to these technologies (137, 140). Demonstrating a clear cost-effectiveness benefit over current standard-of-care approaches will be essential for widespread adoption.

Looking ahead, the vision for precision immuno-oncology in NSCLC is one of dynamic, adaptive, and patient-centered management. Future clinical trials should be designed *a priori* to incorporate these multi-parametric biomarker strategies, using them not only for patient stratification but also for guiding treatment decisions in real-time (116, 135). For instance, ctDNA-based MRD detection could trigger a switch in therapy before radiographic progression is evident (45, 46). A composite biomarker panel integrating genomics, proteomics, and radiomics could identify patients who are likely to benefit from ICI monotherapy versus those who require more intensive combination strategies (73, 135). The integration of emerging frontiers, such as the microbiome and epigenome, will further refine our understanding and predictive capabilities (129, 132). Concurrently, the rapid evolution of interdisciplinary innovations—ranging from sophisticated analytical platforms to novel data-driven diagnostic frameworks—provides a strong foundation for overcoming existing translational barriers (141–143). Ultimately, the goal is to move beyond a one-size-fits-all approach and deliver the right immunotherapy to the right patient at the right time, maximizing the chance of durable benefit while minimizing unnecessary toxicity and cost. The journey from a single, imperfect biomarker to a dynamic, multi-parametric framework is well underway, and it holds the promise of transforming the landscape of NSCLC immunotherapy for years to come.
